# Assessment of longitudinal distribution of subclinical atherosclerosis in femoral arteries by three-dimensional cardiovascular magnetic resonance vessel wall imaging

**DOI:** 10.1186/s12968-018-0482-7

**Published:** 2018-09-03

**Authors:** Yongjun Han, Maobin Guan, Zhu Zhu, Dongye Li, Huijun Chen, Chun Yuan, Cheng Li, Wei Wang, Xihai Zhao

**Affiliations:** 1 Center for Brain Disorders Research, Capital Medical University and Beijing Institute of Brain Disorders, Beijing, China; 2 Center for Biomedical Imaging Research, Department of Biomedical Engineering, Tsinghua University School of Medicine, Beijing, China; 3 Department of Radiology, The Affiliated Hospital of Yangzhou University, Yangzhou University, Yangzhou, China; 4 Department of Radiology, University of Washington, Seattle, USA; 5 Department of Radiology, Zhongda Hospital, Medical School of Southeast University, Nanjing, China; 6 Center of Stroke, Beijing Institute for Brain Disorders, Beijing, China

**Keywords:** Femoral artery, Atherosclerosis, Peripheral artery disease, Distribution, Magnetic resonance imaging

## Abstract

**Background:**

Lower extremity peripheral artery disease has become a significant health burden worldwide. Since the treatment strategies can be different if atherosclerotic disease involves different femoral artery segments, it is important to assess plaque distribution among different segments of femoral arteries. We sought to investigate the longitudinal distribution of subclinical femoral artery atherosclerosis in asymptomatic elderly adults using cardiovascular magnetic resonance (CMR) vessel wall imaging.

**Methods:**

Asymptomatic elderly subjects underwent three-dimensional (3D) CMR vessel wall imaging for femoral arteries. The 3D motion sensitized-driven equilibrium prepared rapid gradient-echo (3D-MERGE) sequence was acquired from the common femoral artery to the popliteal artery. The femoral artery was divided into 4 segments: common femoral artery (CFA), proximal superficial femoral artery (pSFA), adductor canal (AC) segment of femoral artery, and popliteal artery (PA). The morphological characteristics including lumen area, wall area, maximum and minimum wall thickness, normalized wall index (NWI = wall area / [lumen area + wall area] × 100%), and eccentricity index ([maximum wall thickness - minimum wall thickness] / maximum wall thickness), luminal stenosis, and presence of atherosclerotic plaque were evaluated and compared between bilateral sides and among different femoral artery segments in each side of femoral artery. The associations between ankle-brachial index (ABI) and cardiovascular risk factors and femoral artery plaque characteristics were also determined.

**Results:**

Of 107 recruited subjects (71.9 ± 5.6 years; 48 males), 70 (65.4%) were found to have femoral artery plaques. The atherosclerotic plaques were most frequently found in PA (41.1%) and CFA (40.2%) segments, followed by pSFA (31.8%) and AC (23.4%) segments (*p* = 0.002). Similarly, PA and CFA segments showed significantly greater maximum wall thickness and eccentricity index compared with pSFA and AC segments (all *p* < 0.001). Significant differences can be found in NWI among four segments of femoral arteries (*p* < 0.001) and PA showed the highest NWI (54.8%), followed by AC (54.3%), pSFA (52.4%) and CFA (45.9%) segments. Compared with right femoral artery, left femoral artery had significant smaller lumen area and greater NWI in most of segments (*p* < 0.002). There were no significant differences in ABI between subjects with and without atherosclerotic plaques (*p* = 0.161). The presence of subclinical atherosclerotic plaque in femoral arteries was significantly associated with cardiovascular risk factors including age (odds ratio [OR], 1.133; 95% confidence interval [CI], 1.048–1.224, *p* = 0.002), male gender (OR, 3.914; 95% CI, 1.612–9.501, *p* = 0.003), and hypertension (OR, 4.000; 95% CI, 1.700–9.411, *p* = 0.001), respectively.

**Conclusions:**

Subclinical femoral artery atherosclerosis is prevalent in the elderly population, particularly in the left femoral artery and segments of CFA and PA, and is associated with age, male gender and hypertension. Our findings suggest that, for screening subclinical atherosclerosis, more attention needs to be paid to the specific side and segments of femoral arteries, particularly older individuals and those with these cardiovascular disease risk factors.

**Electronic supplementary material:**

The online version of this article (10.1186/s12968-018-0482-7) contains supplementary material, which is available to authorized users.

## Background

As a manifestation of systemic atherosclerotic disease, lower extremity peripheral artery disease (LE-PAD) has become a significant health burden worldwide, particularly in the middle-aged and elderly populations [1–3]. LE-PAD can be progressed from subclinical stage to clinically symptomatic disease and subsequently lead to reduction in functional capacity of life and limb amputation. There is increasing evidence that patients with LE-PAD are at high risk of developing cardiovascular events, including myocardial infarction and stroke [4, 5]. Since the longitudinal coverage of femoral arteries reaches around 50 cm from common femoral artery (CFA) to the popliteal artery (PA) segments, the treatment strategies can be different if atherosclerotic disease involves different femoral artery segments. Therefore, it is important to assess the atherosclerotic plaque distribution among different segments of femoral arteries for precise diagnosis, patient management and event prevention.

Clinically, ankle-brachial index (ABI) has been widely used to diagnose LE-PAD. However, investigators found that substantial atherosclerosis can be present in the femoral arteries of patients with a normal ABI [6, 7]. Although ultrasound imaging is a non-invasive imaging modality, the operator-dependent results and poor reproducibility limit its application in precise diagnosis, especially for characterizing plaque compositional features. Recently, three-dimensional (3D) cardiovascular magnetic resonance (CMR) vessel wall imaging techniques have been proposed for evaluating atherosclerotic plaques in carotid [8] and femoral arteries [9–11]. Benefiting from the iso-tropic high spatial resolution and large longitudinal coverage, 3D vessel wall imaging techniques have been successfully utilized for assessing plaque distribution in intracranial and extracranial carotid arteries [12, 13]. We sought to investigate the distribution of subclinical atherosclerotic diseases among different femoral artery segments in the elderly population using 3D CMR vessel wall imaging.

## Methods

### Study sample

All subjects were recruited from a pilot community study of Cardiovascular Risk in Old Population (CROP). The inclusion criteria are as follows: 1) age ≥ 60 years; 2) no cardiovascular symptoms within 6 months; and 3) with or without history of coronary heart disease or stroke. Subjects with heart failure, history of LE-PAD, or contraindications to CMR examination were excluded. All the participants underwent 3D CMR vessel wall imaging for bilateral femoral arteries. Clinical characteristics including age, gender, body mass index (BMI), history of smoking (current or former), diabetes (fasting blood sugar level ≥ 126 mg/dL, 2-h oral glucose tolerance test result ≥200 mg/dL, or hemoglobin A1c ≥6.5%), hypertension (diastolic blood pressure ≥ 90 mmHg or systolic blood pressure ≥ 140 mmHg), hyperlipidemia (elevated concentrations of any or all of the following lipids in the plasma: low density lipoprotein > 140 mg/dL, total cholesterol > 200 mg/dL, or triglycerides > 150 mg/dL), and coronary heart disease were collected from the clinical record. For each subject, the ABI was measured 3 times at supine position on resting state and the average value was recorded. The study protocol was approved by local Institutional Review Board and written informed consent was obtained from all subjects.

### CMR imaging protocol

**Fig. 1:**
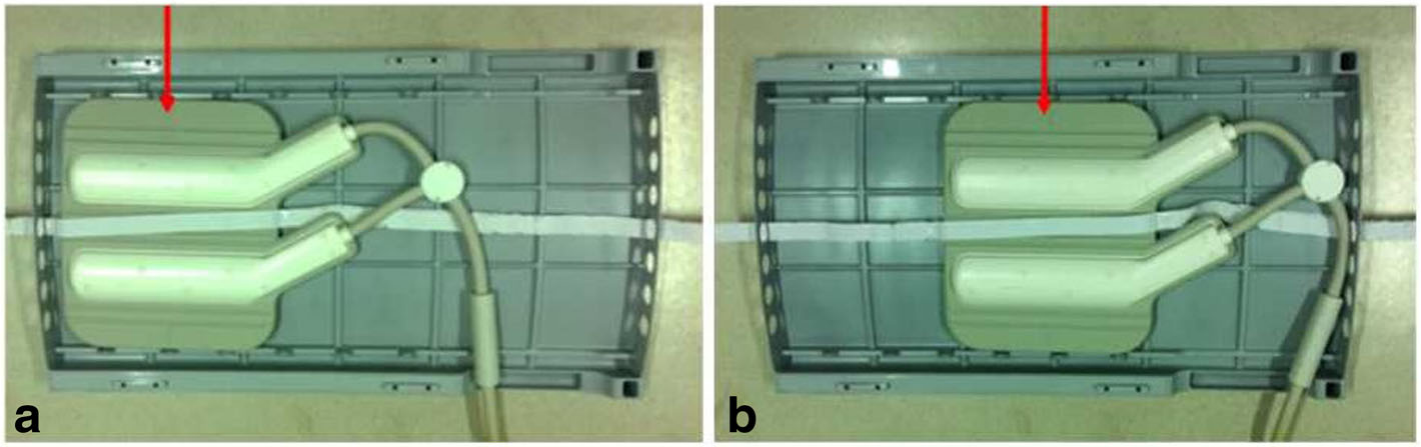
The pictures of the back side of supporting box. **a** The location of the back elements of cardiac coil (red arrow) when acquired the CMR images of femoral artery at the upper region. Before the acquisition of CMR images of femoral artery at the lower region, the back elements of cardiac coil (red arrow) were sliding from the upper to the lower regions along z axis by pulling the rope (**b**)

CMR vessel wall imaging for bilateral femoral arteries was performed on a 3 T scanner (Achievia TX, Philips Healthcare, Best, The Netherlands) with a 32-channel phased-array heart coil. A 3D motion sensitized-driven equilibrium prepared rapid gradient-echo (3D-MERGE) imaging sequence was acquired using the following parameters: fast field echo sequence, repetition time/echo time 9.1/4.2 ms, flip angle 6°, field of view 300 × 400 × 60 mm^3^, spatial resolution 0.8 × 0.8 × 0.8 mm^3^. To facilitate CMR vessel wall imaging covering from the CFA to the PA, two stacks of CMR imaging were conducted with 40% overlap longitudinally using a custom-designed supporting box (Fig. 1) which was placed between human body and the back elements of coil. By using the supporting box, the back elements of cardiac coil can be sliding from the upper to the lower regions along z axis when acquired the corresponding stack of CMR images without moving the subjects. The average CMR scan time for each subject was 20 min.

### CMR image analysis

**Fig. 2:**
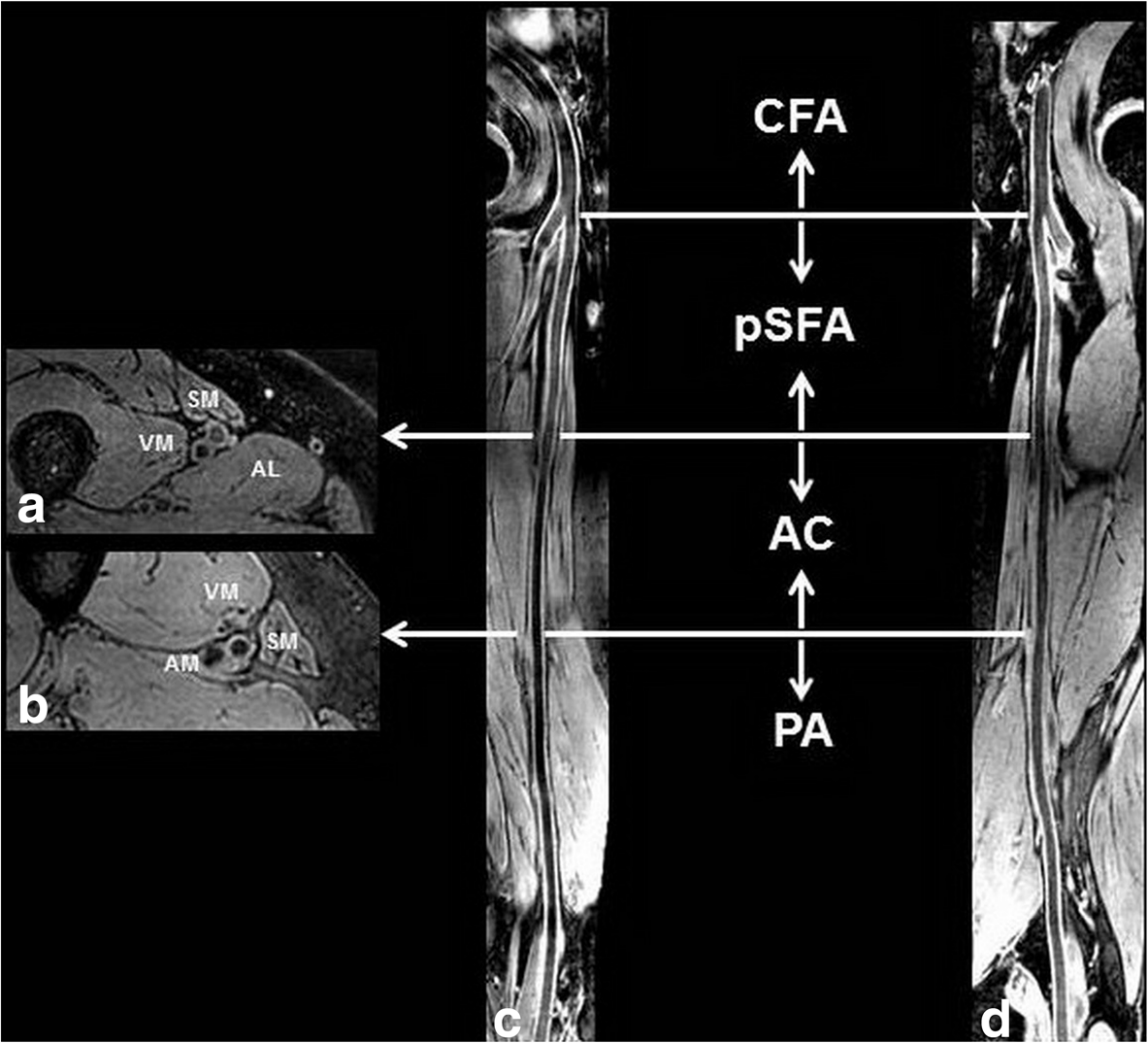
Segments of femoral artery. **a**, **b** normal subject and represent the axial images at the level of the inlet and outlet of adductor canal, respectively. **c**, **d** right and left femoral arteries, respectively. CFA: common femoral artery. pSFA: proximal superficial femoral artery. AC: adductor canal segment. PA: popliteal artery. SM: Sartorius muscle. AL: adductor longus. VM: vastus medialis. AM: adductor magnus

The quality of CMR images was evaluated with 4-point scale (1, poor; 2, marginal; 3, good; and 4, excellent) according to the clearness of boundaries of lumen and outer wall, plaque compositions and presence/absence of artifacts [14]. Subjects with poor image quality were excluded from final analysis. For each subject, two stacks of CMR images were fused on a CMR workstation (Extended MR WorkSpace 2.6.3.4, Philips Healthcare). Two experienced radiologists (> 3 years’ experience in cardiovascular imaging) reviewed the CMR images using a custom-designed software 3D-CASCADE with consensus. The femoral arteries were divided into four segments (Fig. 2): 1) common femoral artery (CFA); 2) proximal of superficial femoral artery (pSFA): from the formal artery bifurcation to the beginning of adductor artery; 3) adductor canal (AC) segment of femoral artery: the segment of SFA in adductor canal; and 4) PA. The centerline of arteries was determined and the CMR data were resliced to cross-sectional images with 2 mm thickness perpendicular to the centerline. The boundaries for lumen and outer wall for each cross-sectional image were semi-automatically outlined. The presence of atherosclerotic plaque is defined as lesions with eccentric wall thickening which represent American Heart Association (AHA) type-III plaques or above according the modified AHA criteria for atherosclerotic plaques on CMR vessel wall imaging [15]. For each femoral artery segment, if there was a plaque, all slices with plaque and the adjacent 3 slices with normal wall were measured. For arterial segments without plaque, the cross-sectional slices were analyzed every other 5 slices. The morphological characteristics including lumen area, wall area, maximum and minimum wall thickness, normalized wall index (NWI = wall area / [lumen area + wall area] × 100%), and eccentricity index ([maximum wall thickness - minimum wall thickness] / maximum wall thickness) were measured and the presence or absence of atherosclerotic plaque which is defined as eccentric wall thickening was identified for each slice. In addition, the luminal stenosis for each arterial segment was measured on the 3D-MERGE images. Previous study demonstrated that there was excellent agreement in measuring carotid artery stenosis between 3D-MERGE images and digital subtraction angiography (intraclass correlation coefficient [ICC], 0.96; 95% confidence interval [CI], 0.93–0.97) [16]. The average time for qualitatively and quantitatively analyzing the MR images for each side of femoral artery was 2 h.

### Reproducibility

Five participants were randomly selected for reproducibility study. In total, 40 arterial segments from 10 femoral arteries were analyzed for testing the inter-reader and intra-reader reproducibility, respectively. For evaluating the inter-reader reproducibility, two reviewers interpreted the CMR images independently. For assessing the intra-reader reproducibility, one reviewer interpreted the CMR images twice with 2 months’ time interval to minimize the memory bias.

### Statistical analysis

The continuous quantitative variables were presented with mean ± standard deviation (SD), and the categorical variables were described as percentage. The mean value (NWI, lumen area, and wall area) or maximum value (maximum wall thickness, eccentric index, and luminal stenosis) of morphological measurements were taken from bilateral femoral arteries for each subject. The morphological measurements and prevalence of atherosclerotic plaque were compared between left and right sides and among different arterial segments (combined bilateral sides) using generalized estimating equations (GEE) analysis. Spearman correlation analysis was performed to evaluate the association of ABI with max wall thickness, eccentricity index, NWI, and luminal stenosis. The correlation between morphological measurements of femoral arteries and cardiovascular risk factors was determined utilized Spearman correlation and multiple correlation analysis. Femoral artery plaque characteristics were compared between male and female subjects using independent *t* test or Chi-square test when appropriate. Logistic regression was utilized to calculate the odds ratio (OR) and corresponding 95% CI of cardiovascular risk factors in discriminating presence of femoral artery plaque. The ABI was compared between patients with and without plaque in femoral arteries with independent *t* test. The ICC and corresponding 95% CI were calculated to assess the intra-reader and inter-reader agreement in measuring femoral artery morphology. All statistical analyses were conducted with SPSS software, version 16.0 (International Business Machines, Armonk, New York, USA). In general, a value of *p* < 0.05 was considered statistically significant. During above multiple comparisons, the level of significance (type-I error rate) was adjusted by Bonferroni test to avoid false positives.

## Results

**Fig. 3:**
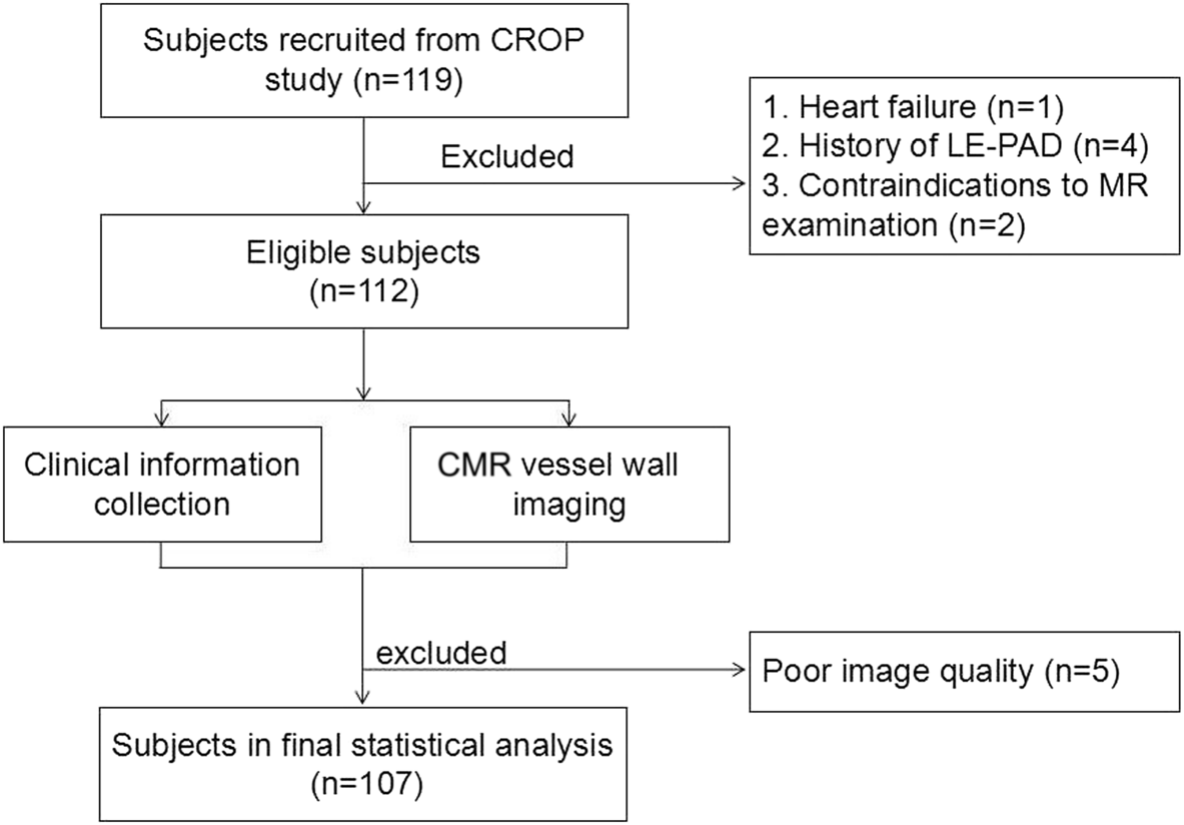
Flow chart for subject recruitment, CROP: Cardiovascular Risk in Old Population; LE-PAD: lower extremity peripheral artery disease

Figure 3 is the flow chart for subject recruitment. Of the 112 recruited subjects, 5 were excluded due to poor CMR image quality. Of the remaining 107 participants (71.9 ± 5.6 years; 48 (44.9%) males), 13 (12.1%) had a history of smoking, 55 (51.4%) had hypertension, 22 (20.6%) had diabetes, 67 (62.6%) had hyperlipidemia, and 18 (16.8%) had coronary heart disease. The clinical characteristics of this study population are summarized in Table 1.

**Table 1:** Clinical characteristics of study population (*n* = 107)

	Mean ± SD or n (%)	Range
Age, years	71.9 ± 5.6	58–84
Gender, male	48 (44.9)	–
Smoking	13 (12.1)	–
Weight, kg	63.4 ± 9.8	40–90
Height, cm	162.9 ± 8.0	148–183
Body mass index, kg/m²	23.8 ± 2.8	15.4–34.2
Hypertension	55 (51.4)	–
Diabetes	22 (20.6)	–
Hyperlipidemia	67 (62.6)	–
High density lipoprotein, mmol/L	1.5 ± 0.4	0.8–2.9
Low density lipoprotein, mmol/L	3.0 ± 0.9	1.3–5.3
Triglycerides, mmol/L	1.6 ± 1.0	0.5–7.9
Total cholesterol, mmol/L	5.0 ± 0.9	3.0–8.0
Ankle-brachial index	1.1 ± 0.1	0.9–1.7
Coronary heart disease	18 (16.8)	–

### CMR imaging characteristics of femoral artery atherosclerosis

**Fig. 4:**
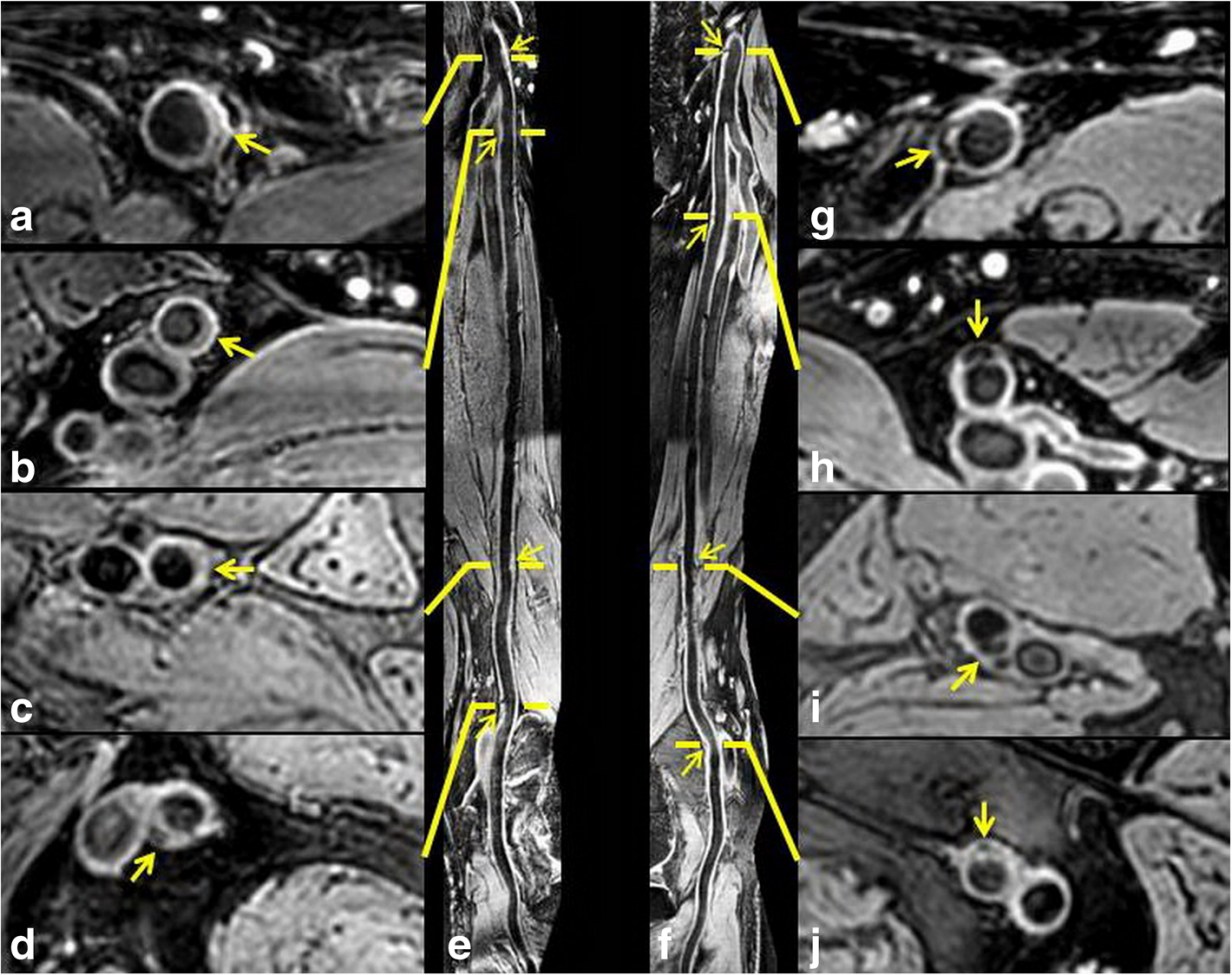
Example for femoral arteries with multiple atherosclerotic plaques. The images are from a 79 years old male patient. Multiple atherosclerotic plaques (yellow arrows) can be found in right (**e**) and left (**f**) femoral arteries. The atherosclerotic plaques (yellow arrows) were clearly delineated on the axial images at the segment of common femoral artery (**a** and **g**), proximal superficial femoral artery (**b** and **h**), adductor canal (**c** and **i**), and popliteal artery (**d** and **j**)

We found that 65.4% of subjects had subclinical femoral artery atherosclerotic plaques. The CMR imaging characteristics of femoral artery atherosclerosis among different segments are presented in Table 2. The atherosclerotic plaques were most frequently found in PA (41.1%) and CFA (40.2%) segments, followed by pSFA (31.8%) and AC (23.4%) segments (*p* = 0.002). Similarly, PA and CFA segments showed significantly greater maximum wall thickness and eccentric index compared with pSFA and AC segments (all *p* < 0.001). The lumen area and wall area were found to be decreased from CFA to PA segments (all *p* < 0.001). Significant differences can be found in normalized wall index among four segments of femoral arteries (*p* < 0.001) and PA showed the highest NWI (54.8%), followed by AC (54.3%), pSFA (52.4%) and CFA (45.9%) segments. There were no significant differences in luminal stenosis among different femoral artery segments (*p* = 0.016). Figure 4 represents an example for a subject with subclinical multiple atherosclerotic plaques in bilateral femoral arteries.

**Table 2:** CMR plaque characteristics among different femoral artery segments

	Mean ± SD or n (%)				p#
	CFA (*n* = 107)	pSFA (*n* = 107)	AC (*n* = 107)	PA (*n* = 107)	
Lumen area, mm²	49.6 ± 11.8	29.4 ± 8.1	25.1 ± 6.4	25.1 ± 6.8	<0.001
Wall area, mm²	41.6 ± 7.6	31.8 ± 5.6	29.4 ± 5.5	30.0 ± 5.3	<0.001
Max wall thickness, mm	2.3 ± 1.0	2.0 ± 0.7	1.9 ± 0.7	2.1 ± 1.0	<0.001
Normalized wall index, %	45.9 ± 4.9	52.4 ± 4.4	54.3 ± 4.6	54.8 ± 4.8	<0.001
Eccentricity index	0.43 ± 0.19	0.38 ± 0.15	0.36 ± 0.16	0.39 ± 0.18	<0.001
Luminal stenosis, %^a^	38.1 ± 14.4	32.6 ± 14.4	36.5 ± 15.1	31.9 ± 11.8	0.016
Presence of plaque	43 (40.2)	34 (31.8)	25 (23.4)	44 (41.1)	0.002

*CFA* common femoral artery, *pSFA* proximal superficial femoral artery, *AC* adductor canal segment, *PA* popliteal artery. ^a^the measurement was calculated from arteries with plaque. #The level of significance (type-I error rate) was less than 0.007 by adjustment of Bonferroni test

### Comparison of atherosclerosis between left and right femoral arteries

The comparison of CMR characteristics of atherosclerosis between left and right femoral arteries is shown in Table 3. The lumen area of left femoral arteries in AC and PA segments were significantly smaller than that of right femoral arteries (all *p* < 0.002). On the contrary, left femoral arteries had significantly greater NWI compared with right femoral arteries in all 4 segments (all *p* < 0.002). Although left femoral arteries showed greater wall area compared with right femoral arteries in all 4 segments, only the differences in CFA and PA segments were statistically significant (both *p* < 0.002). No significant differences can be found in maximum wall thickness, eccentric index, luminal stenosis, and prevalence of plaques between left and right femoral arteries in any segment (all *p* > 0.002).

**Table 3:** Comparison of CMR characteristics of atherosclerosis between left and right femoral arteries

	Mean ± SD or n (%)			
	CFA	pSFA	AC	PA
Lumen area, mm²				
Left	49.0 ± 11.7	28.9 ± 8.1	24.4 ± 6.4	24.3 ± 6.3
Right	50.3 ± 12.0	29.9 ± 8.2	25.6 ± 6.5	25.7 ± 7.6
p#	0.010	0.002	<0.001	<0.001
Wall area, mm²				
Left	43.4 ± 8.4	32.4 ± 5.9	29.7 ± 5.5	31.2 ± 6.0
Right	41.6 ± 7.9	31.8 ± 5.8	29.5 ± 5.5	29.9 ± 5.5
p#	0.002	0.438	0.240	0.001
Normalized wall index, %				
Left	47.3 ± 5.1	53.4 ± 4.9	55.3 ± 0.5	56.5 ± 4.8
Right	45.6 ± 5.5	52.1 ± 4.3	53.7 ± 0.5	54.3 ± 5.5
p#	<0.001	<0.001	<0.001	<0.001
Maximum Wall thickness, mm				
Left	2.4 ± 1.0	2.1 ± 0.7	1.94 ± 0.7	2.2 ± 0.8
Right	2.2 ± 1.0	2.0 ± 0.7	1.91 ± 0.7	2.1 ± 1.1
p#	0.028	0.284	0.573	0.938
Eccentricity index				
Left	0.4 ± 0.2	0.4 ± 0.1	0.4 ± 0.2	0.4 ± 0.2
Right	0.4 ± 0.2	0.4 ± 0.2	0.4 ± 0.2	0.4 ± 0.2
p#	0.263	0.144	0.588	0.529
Luminal stenosis, %^a^				
Left	39.1 ± 14.0	37.5 ± 16.7	33.8 ± 10.7	36.9 ± 10.1
Right	36.5 ± 20.7	34.0 ± 15.4	36.4 ± 12.7	36.7 ± 12.8
p#	0.901	0.284	0.207	0.789
Presence of plaque				
Left	34 (31.8)	26 (24.5)	16 (15.2)	32 (30.5)
Right	28 (26.2)	19 (17.9)	17 (16.2)	30 (28.6)
p#	0.219	0.231	0.809	0.848

*CFA* common femoral artery, *pSFA* proximal superficial femoral artery, *AC* adductor canal segment, *PA* popliteal artery. ^a^the measurement was calculated from arteries with plaque. #The level of significance (type-I error rate) was less than 0.002 by adjustment of Bonferroni test

### Associations of CMR morphology with ABI and cardiovascular risk factors

All subjects had an ABI > 0.9. Spearman’s correlation analysis showed a weak negative correlation between ABI and NWI (*r* = − 0.336, *p* = 0.001). No significant correlation between ABI and luminal stenosis was found (*r* = 0.024, *p* = 0.855). There were no significant differences in ABI between subjects with and without atherosclerotic plaques (1.12 ± 0.10 vs. 1.14 ± 0.12, *p* = 0.161). Table 4 summarized the results on the correlation between plaque measurements and cardiovascular risk factors. There were significant correlations between maximum wall thickness and age (*r* = 0.211, *p* = 0.029), male gender (0.202, *p* = 0.037), hypertension (*r* = 0.305, *p* = 0.001), and hyperlipidemia (*r* = 0.202, *p* = 0.042). Significant correlations were found between presence of plaque and age (*r* = 0.308, *p* = 0.001), male gender (*r* = 0.300, *p* = 0.002), and hypertension (*r* = 0.315, *p* = 0.001). Similar correlations were also found between eccentricity index and age, male gender, and hypertension (all *p* < 0.05). In addition, the NWI was found to be significantly correlated with male gender (*r* = − 0.332, *p* < 0.001) but not with other risk factors (all *p* > 0.05). The multiple correlation analysis showed that, after adjusted for confounding factors, the correlation of plaque presence with age (*r* = 0.216, *p* = 0.029), gender (*r* = 0.246, *p* = 0.013), and triglycerides (*r* = 0.223, *p* = 0.024) and the correlation of eccentricity index with gender (r = 0.202, *p* = 0.041) and triglycerides (*r* = 0.295, *p* = 0.003) remained statistically significant, respectively. Compared to female subjects, male ones had significantly greater lumen area, wall area, eccentricity index, and prevalence of plaque but smaller NWI (all *p* < 0.05, Additional file 1: Table S1). No significant correlations can be found between femoral artery morphology and BMI, smoke, diabetes, and coronary heart disease (all *p* > 0.05). Logistic regression analysis revealed that the OR for age, male gender, and hypertension was 1.133 (95% CI, 1.048–1.224, *p* = 0.002), 3.914, (95% CI, 1.612–9.501, *p* = 0.003), and 4.000, (95% CI, 1.700–9.411, *p* = 0.001) in discriminating presence of femoral artery plaque, respectively.

**Table 4:** Correlation between plaque measurements and cardiovascular risk factors

	Plaque characteristics on CMR imaging									
	Presence of plaque		Max WT		Eccentricity index		NWI		Luminal stenosis	
	r	p	r	p	r	p	r	p	r	p
Age, years	0.308	0.001	0.211	0.029	0.221	0.022	0.116	0.223	−0.096	0.452
Gender, male	0.300	0.002	0.202	0.037	0.246	0.011	−0.332	<0.001	−0.084	0.507
BMI, kg/m²	0.168	0.083	0.167	0.085	0.162	0.096	−0.111	0.255	−0.063	0.622
Smoke	0.090	0.357	0.054	0.579	0.101	0.301	0.022	0.820	0.041	0.746
Hypertension	0.315	0.001	0.305	0.001	0.320	0.001	0.145	0.135	0.154	0.224
Diabetes	0.135	0.170	0.057	0.565	0.071	0.474	0.100	0.308	0.033	0.799
Hyperlipidemia	0.162	0.103	0.202	0.042	0.166	0.096	−0.039	0.698	0.206	0.108
HDL, mmol/L	−0.102	0.304	−0.142	0.150	−0.167	0.090	−0.033	0.740	−0.213	0.097
LDL, mmol/L	0.129	0.192	0.082	0.410	0.061	0.537	−0.077	0.435	−0.077	0.553
TG, mmol/L	0.200	0.040	0.303	0.002	0.281	0.004	0.123	0.213	0.218	0.089
TC, mmol/L	0.190	0.054	0.173	0.079	0.149	0.132	0.080	0.418	0.007	0.955
CHD	−0.018	0.865	−0.044	0.668	0.016	0.876	0.121	0.237	−0.133	0.327

*BMI* Body mass index, *HDL* High density lipoprotein, *LDL* Low density lipoprotein, *TG* Triglycerides, *TC* Total cholesterol, *CHD* Coronary heart disease, *ABI* Ankle-brachial index, *Max WT* Maximum wall thickness, *NWI* Normalized wall index

### Reproducibility

Excellent intra-reader (ICC, 0.97; 95% CI, 0.94–0.97, *p* < 0.001) and inter-reader (ICC, 0.98; 95% CI, 0.97–0.98, *p* < 0.001) agreement was found in measuring femoral artery maximum wall thickness. In identification of atherosclerotic plaques, the inter-reader agreement was excellent (kappa value, 0.94; *p* < 0.001).

## Discussion

This study investigated the characteristics of femoral artery subclinical atherosclerotic plaques, particularly the longitudinal distribution among different segments, and their relationships with ABI in asymptomatic elderly adults using CMR vessel wall imaging. We found that PA and CFA segments had the highest incidence of atherosclerotic plaques and plaque burden among all femoral artery segments. No significant correlation was found between presence of femoral artery plaque and ABI and more than 60% subjects with normal ABI had atherosclerotic plaques in femoral arteries.

Atherosclerotic plaques were frequently seen in femoral arteries of asymptomatic elderly subjects in the present study. A recent ultrasound imaging study investigating the same elderly population to the present study documented a similar prevalence of subclinical femoral artery atherosclerosis (57%) [17]. High prevalence of subclinical femoral artery atherosclerosis determined by ultrasound has also been reported in the middle-aged Spain populations (prevalence: 44–54%) [1, 3]. Clinically, ABI defined as the ratio of the blood pressure at the ankle to the blood pressure in the upper arm is the most common metrics for measuring femoral artery atherosclerosis with cutoff point of ABI < 0.9. However, in the present study, the ABI of all subjects was > 0.9 and there were no significant differences in ABI between patients with and without femoral artery plaques. Our results indicate that CMR vessel wall imaging is superior to ABI in detecting subclinical femoral artery atherosclerosis.

In the present study, atherosclerotic plaquewas found to be more prevalent and severe in PA and CFA segments among all femoral artery segments. The high incidence of atherosclerotic plaques in these segments can be explained by the specific hemodynamic characteristics prior to the bifurcation. Previous studies have shown that the changes of blood flow direction and velocity due to the flow diverter play important role in initiation of atherosclerosis [18]. This phenomenon has been largely discussed in carotid arteries [19, 20]. Different from our findings, Chi et al. [21] demonstrated that the maximum wall thickness in adductor segment was significantly greater than that of the bifurcation area of femoral artery (3.56 ± 1.33 mm vs. 2.83 ± 1.07 mm, *p* = 0.0021) in patients with intermittent claudication. Investigators believed that the AC segment of femoral artery is susceptible to having severe atherosclerosis because this arterial segment is surrounded by four muscles which may restrict the compensatory outward enlargement during plaque progression. Our findings suggest that more attention needs to be paid to common femoral artery and PA segments of femoral arteries in assessing the atherosclerotic disease in asymptomatic subjects.

We found that left femoral arteries had significantly greater burden of atherosclerotic plaque as measured by NWI compared with right femoral arteries. The symmetry of atherosclerotic diseases in bilateral femoral arteries in previous studies was controversial. Wikström et al. demonstrated that significant association between right and left of superficial femoral arteries (*p* = 0.0058) and popliteal arteries (*p* = 0.16) in an unselected elderly population with 50% stenosis on CMR angiography [22]. Similarly, a study by Vink et al. reported that the size (r^2^ = 0.5, *p* < 0.001), remodeling (kappa = 0.42, *p* = 0.007), and vulnerability (large lipid-rich core, kappa = 0.60, *p* = 0.001) of femoral plaques were symmetric in bilateral femoral arteries [23]. On the contrary, a previous study recruited 2524 healthy subjects (age range 35–55 years) showed significant differences between left and right femoral arteries in intima-media thickness distribution (right: 1.11 mm, left 1.01 mm; *p* < 0.001) and presence of plaque (right: 21.9% vs. left: 15.7%; *p* < 0.001) by ultrasonographic examination [24]. In contrast, our study showed that left femoral arteries had more severe atherosclerotic disease than right femoral arteries. Above inhomogeneous results on the symmetry of bilateral femoral artery atherosclerosis might be due to the different study populations and methodologies.

We found that femoral artery morphological characteristics measured by CMR imaging, such as maximum wall thickness and eccentricity index, and presence of plaque were significantly associated with cardiovascular risk factors. Our findings are consistent with previous studies. A number of studies demonstrated that there were significant correlations between femoral artery atherosclerosis and cardiovascular risk factors [25, 26]. A study by Kirhmajer et al. reported that femoral intima-media was correlated with BMI (*r* = 0.16, *p* = 0.036), high-density lipoprotein (*r* = − 0.30, *p* = 0.0003), and triglycerides (*r* = 0.19, *p* = 0.17) [26]. An AWHS study showed that the presence of subclinical femoral atherosclerotic plaque was associated with cardiovascular risk factors, such as dyslipidemia (OR, 1.46; 95% CI, 1.17–1.83), hypertension (OR, 1.66; 95% CI, 1.31–2.10), current smoking (OR, 3.88; 95% CI, 3.01–5.00), and diabetes (OR, 2.11; 95% CI, 1.20–3.70) [3].

3D CMR vessel wall imaging techniques were utilized to characterize femoral artery atherosclerosis in this study. In the present study, 3D MERGE imaging sequence was acquired to provide high resolution morphological information of arterial wall. This technique has been largely used for evaluating carotid artery [12, 13, 27] and femoral artery atherosclerotic plaques [21]. In delineating femoral artery atherosclerosis, other 3D imaging techniques such as variable flip angle turbo spin echo (SPACE) [11] and delay alternation with nutation for tailored excitation (DANTE-FLASH) [10] imaging sequences have been utilized as well. Since the longitudinal coverage of femoral arteries is around 50 cm, the current available coil could not facilitate it at one scan section. To address this issue, we designed a supporting box and the coil can be freely sliding from upper location to the lower location without moving patient. To design a lower extremity artery dedicated coil with large longitudinal coverage and sufficient elements for high resolution vessel wall imaging is warranted in future studies.

### Limitations

Our study has several limitations. First, we only evaluated the plaque morphology of femoral artery atherosclerosis. It will be interesting to assess the femoral artery plaque compositional features in future studies. Second, this study focused on the elderly population. We would recruit subjects with more broad range of age in future studies. Third, this is a cross-sectional study and the relationship between the morphology of subclinical femoral artery atherosclerosis measured by CMR vessel wall imaging and cardiovascular events is unclear. Future prospective studies with long term follow-up are suggested.

## Conclusions

Subclinical femoral artery atherosclerosis is prevalent in the elderly population, particularly in the left femoral artery and segments of common femoral artery and popliteal artery, and its associated risk factors include age, male gender and hypertension. Our findings suggest that, for screening subclinical atherosclerosis, more attention needs to be paid to the specific side and segments of femoral arteries, particularly for elderly individuals with these cardiovascular risk factors.

## Additional file

Additional file 1: **Table S1.** Comparison of femoral artery plaque features between male and female subjects. (DOC 35 kb)

## Abbreviations

3D: three dimensional
AC: Adductor canal
ABI: Ankle-brachial index
AHA: American Heart Association
BMI: Body mass index
CFA: Common femoral artery
CMR: Cardiovascular magnetic resonance
CROP: Cardiovascular Risk in Old Population
GEE: Generalized estimating eqs.
LE-PAD: Lower extremity peripheral artery disease
MERGE: Motion sensitized-driven equilibrium prepared rapid gradient-echo
NWI: Normalized wall index
PA: Popliteal artery
pSFA: Proximal superficial femoral artery
