# Supplementary material for: Assessment of longitudinal distribution of subclinical atherosclerosis in femoral arteries by three-dimensional cardiovascular magnetic resonance vessel wall imaging
Source: J Cardiovasc Magn Reson. 2018 Sep 3;20:60. doi: 10.1186/s12968-018-0482-7 (PMC6120082; doi:10.1186/s12968-018-0482-7)
Supplement: Supplementary file 1 — Table S1. Comparison of femoral artery plaque features between male and female subjects. (DOC 35 kb) [file 12968_2018_482_MOESM1_ESM.doc]

**Table S1** Comparison of femoral artery plaque features between male and femoral subjects.

|  | Mean ± SD, or n (%) | | p |
| --- | --- | --- | --- |
|  | Male  (n=48) | Female  (n=59) |  |
| Lumen area, mm^2^ | 37.0 ± 7.4 | 28.6 ± 5.2 | <0.001 |
| Wall area, mm^2^ | 35.9 ± 4.7 | 31.0 ± 4.6 | <0.001 |
| Max wall thickness, mm | 3.3 ±1.0 | 3.0 ± 1.4 | 0.215 |
| Normalized wall index, % | 50.4 ± 3.7 | 53.1 ± 3.6 | <0.001 |
| Eccentricity index | 0.63 ± 0.14 | 0.55 ± 0.16 | 0.009 |
| Luminal stenosis, % | 42.8 ± 16.1 | 45.2 ± 14.2 | 0.536 |
| Presence of plaque | 39 (81.2) | 31 (52.5) | 0.002 |
